# Dynamic triage in hypertension: a quantitative framework for follow-up scheduling based on clinician heuristics and home blood pressure data

**DOI:** 10.3389/fdgth.2026.1858572

**Published:** 2026-07-21

**Authors:** Jair Andrade, Derek T. O'Keeffe, Ian McCabe, Jennifer Doran, David Tiernan, Andrew J. Simpkin

**Affiliations:** 1 School of Mathematical and Statistical Sciences, University of Galway, Galway, Ireland; 2 HIVE Lab, School of Medicine, University of Galway, Galway, Ireland

**Keywords:** appointment scheduling, blood pressure monitoring, clinical heuristics, decision support, digital health, dynamic triage, hypertension, linear mixed model

## Abstract

**Background:**

Hypertension affects about a third of the global population and stays poorly controlled in many patients despite effective treatments being available. Clinical inertia, meaning the failure to intensify or reassess therapy when it is indicated, is one contributor. Home blood pressure (BP) monitoring and other mobile health (mHealth) tools generate frequent readings, but this adds to the interpretive work asked of clinicians. How clinicians turn home BP readings into follow-up scheduling decisions has had little empirical study.

**Objective:**

To elicit and characterise clinician scheduling preferences for follow-up appointments in newly diagnosed hypertensive patients, using simulated home BP data, as a first step toward mHealth-based scheduling support.

**Methods:**

An online questionnaire presented 15 simulated clinical scenarios depicting newly diagnosed hypertensive patients with 7 days of home BP measurements, and was distributed to physicians in two rounds. Physicians indicated their preferred timing for follow-up (0–5 weeks). We analysed 555 scheduling decisions from 37 physicians using a linear mixed-effects model with a random intercept by physician, which accounts for within-physician correlation.

**Results:**

On the same data used for model fitting, predictions correlated with stated decisions (Pearson *r* = 0.83; median absolute difference 0.1 weeks). This is an internal goodness-of-fit measure and not out-of-sample validation. The most recent follow-up BP was the dominant driver of scheduling. Above the 140 mmHg systolic threshold, higher BP was associated with earlier follow-up (0.13 weeks earlier per 1 mmHg increase; 95% CI 0.09–0.17). Consultants and non-consultants differed: non-consultants scheduled later follow-up at lower BP but earlier follow-up at higher BP.

**Conclusions:**

In simulated scenarios, clinicians appear to use a small set of interpretable rules, chiefly the recent BP value relative to 140 mmHg, when stating preferences for follow-up timing in newly diagnosed hypertensive patients. The formalised rules show meaningful variation between clinicians and give a methodological basis for mHealth scheduling tools. Because the scenarios were simplified and the model has not been tested out of sample, the findings should be read as hypothesis-generating. Whether algorithm-assisted scheduling improves clinical outcomes or reduces workload needs prospective evaluation in real-world settings.

## Background

Elevated blood pressure (BP) is the world's leading modifiable cause of cardiovascular disease and affects about a third of the global population (1). Effective treatments exist (2, 3), yet millions of deaths are still attributed to hypertension each year (4). Only around half of those treated reach adequate control (1). The reasons are well known. They include patient non-adherence, organisational failures, and clinical inertia, which is the lack of treatment intensification or reassessment when this is appropriate (5).

Trial evidence supports intensified pharmacotherapy for BP control (5, 6), but clinical inertia persists in routine practice (7, 8). Physician factors, patient factors, and office systems have all been implicated (7, 9). One proposed remedy is to increase the frequency of outpatient visits (7, 10) so that ordinary care more closely resembles the structured environment of a clinical trial. This is impractical at scale. It strains services and burdens patients, especially those in rural areas or with reduced mobility.

Mobile health (mHealth) (11), which uses sensors and connected devices, offers another route to closer monitoring (12). A prospective study (13) and a randomised trial (5) have both reported that home BP telemonitoring with remote management is associated with significant BP reduction. In the trial, physicians reviewed BP readings monthly and adjusted medication as needed.

Regular review by clinicians adds to workload. It cannot be partly or fully automated without first understanding how clinicians make scheduling decisions. Hypertension guidelines (2) specify a maximum follow-up window of two months for newly diagnosed patients but allow physicians to vary this with disease severity and comorbidity. That flexibility implies implicit heuristics that have not been described formally. Characterising these decision rules is a necessary step before algorithmic support can be built.

A wider literature exists on data-driven follow-up scheduling and decision support across non-communicable diseases. Risk-stratified scheduling models have been described in chronic kidney disease (14), and in diabetes care continuous glucose monitoring and HbA1c-based pathways have been used to vary review frequency (15). Telemonitoring-based decision support has been evaluated in heart failure and in chronic respiratory disease, where remote review of physiological data is used to adjust the timing of clinical contact. Hypertension-specific scheduling models remain sparse. The present study sits within this broader landscape but focuses on newly diagnosed hypertension, where the timing of the first follow-up review matters for medication titration.

## Objectives

We set out to identify which home BP-derived features drive physician scheduling preferences in newly diagnosed hypertension, to describe variation in those preferences between clinicians, and to quantify the goodness-of-fit between a parsimonious model and elicited clinician choices, as a formative step toward mHealth scheduling support.

## Methods

### Ethical considerations

The study was approved by the University Hospital Galway Research Ethics Committee. Participation was voluntary, and physicians gave informed consent before completing the questionnaires. Responses were collected and analysed anonymously. No patient data were used; all clinical scenarios were simulated.

### Study design

The most direct approach would be to analyse real scheduling decisions from patients with hypertension and repeated BP measurements. Such data are not readily available in Ireland because there is no unified system for accessing electronic patient records. We therefore used a vignette-based design with simulated scenarios presented to practising physicians. We prepared a single online questionnaire of 15 scenarios, each depicting a newly diagnosed hypertensive patient, and distributed it to physicians in two rounds. All respondents saw the same 15 scenarios in the same order. The complete set of scenarios is provided in Supplementary Figure S1.

### Scenario development

Each simulated patient had a recent diagnosis of hypertension [systolic BP greater than 135 mmHg (2)], had started antihypertensive medication, and had been given a home BP monitor. The monitor was simulated to record seven consecutive daily BP measurements 2 weeks after diagnosis and treatment initiation, corresponding to the window in which an initial medication effect is expected.

**Figure 1:**
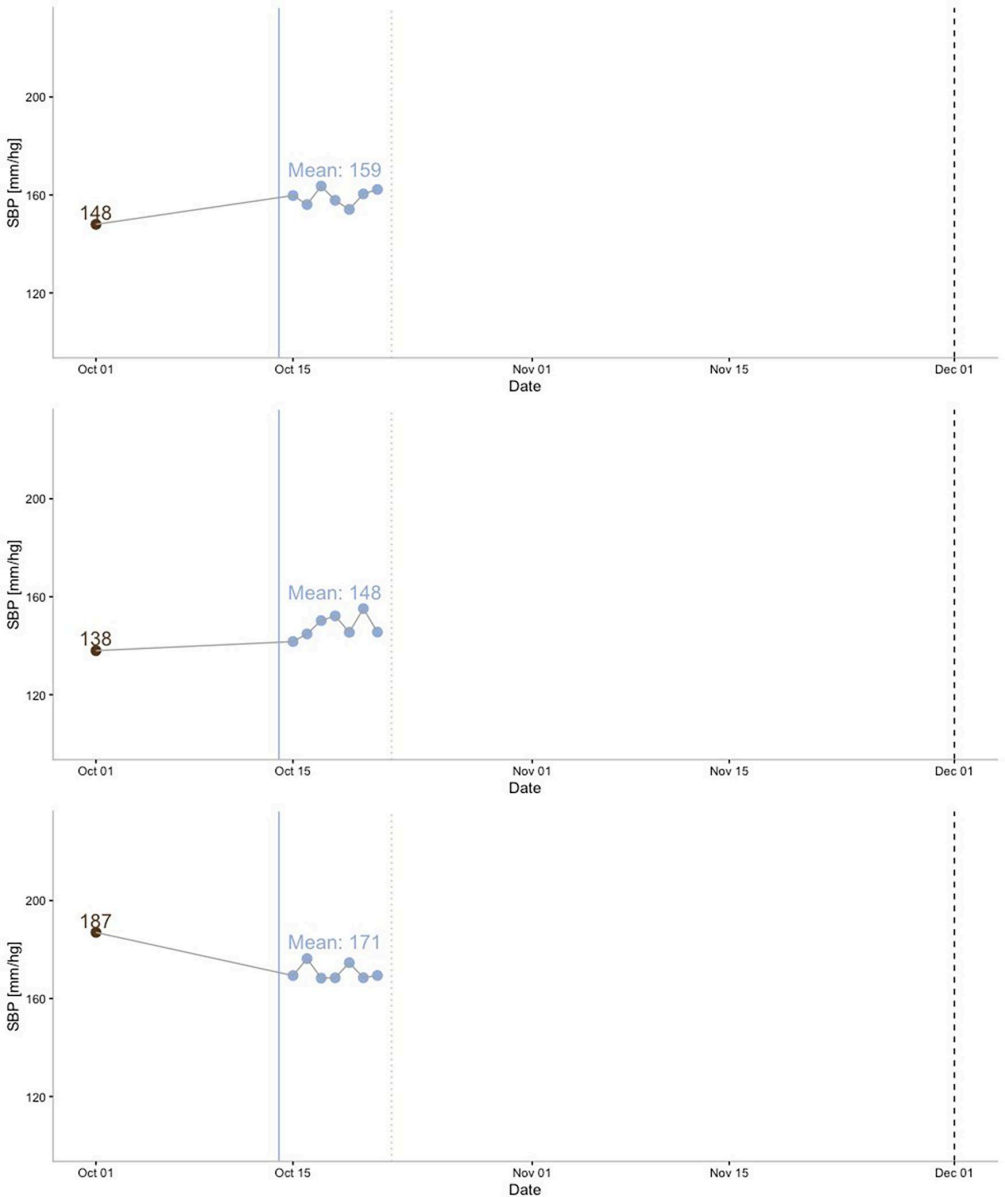
Three representative scenarios from the questionnaire set. In each panel the brown point is the initial diagnostic BP reading and the blue points are the seven consecutive daily home BP measurements recorded 2 weeks after diagnosis and treatment initiation. The three scenarios depict a patient who has reached target, a patient with mildly elevated post-treatment BP, and a patient with persistent severe hypertension. Physicians were asked when they would schedule follow-up review (0–5 weeks). The complete set of scenarios is shown in Supplementary Figure S1.

Scenarios were constructed by varying four design parameters: the initial diagnostic systolic BP, the mean of the seven post-treatment home BP measurements, the day-to-day variability of those measurements (modelled as Gaussian noise with a fixed standard deviation), and the direction of change (improving vs. not improving relative to diagnosis). Across the 15 scenarios, the diagnostic systolic BP ranged from 136 to 187 mmHg and the mean of the seven follow-up home BP measurements ranged from 134 to 171 mmHg. The combinations were chosen to span the clinically meaningful range, and included patients who had reached target, patients who remained mildly elevated, and patients with persistent or worsening hypertension. No comorbidity, medication side-effect, prior-treatment, demographic, or preference data were displayed. The scenarios were reduced to the BP information that a clinician would have when reviewing home monitoring data. Figure 1 shows three representative scenarios from the set, and the complete set is provided in Supplementary Figure S1.

After the last measurement, the patient was due a follow-up appointment. Physicians were asked, based on the BP information shown and the patient's overall wellbeing as presented, to indicate in weeks when they would wish to see the patient. The available options ran from 0 (see the patient immediately) to 5 (see the patient in 5 weeks). Physicians also recorded their professional category (consultant or non-consultant). In the Irish and UK healthcare systems, “consultant” denotes a senior independent practitioner equivalent to the US term “attending physician,” while “non-consultant hospital doctor” (NCHD) is an umbrella term for trainee grades (intern, senior house officer, registrar, and specialist registrar). This terminology is kept throughout for fidelity to the source data; readers in other systems may read it as the experienced vs. in-training distinction.

The scenarios were deliberately simplified. They did not include comorbidity, medication side-effect profiles, prior treatment history, patient preferences, clinic capacity, or any of the contextual information that would normally enter a real consultation. This was a methodological choice to isolate the influence of recent BP data on stated scheduling preferences. It is not a claim that BP data are the only relevant input in practice. The consequences for ecological validity are addressed in the Limitations section. The survey instrument, including the wording of the prompt shown to respondents, is provided as Supplementary Material S2.

### Data collection

The questionnaire was distributed to networks of physicians in two rounds. Twenty-two doctors completed it in the first round and 19 in the second. Four physicians were excluded before analysis on pre-specified quality-control grounds. Three returned the same scheduling response to all 15 scenarios, which gives zero within-respondent variance and indicates that the scenarios were not engaged with individually. One selected the maximum 5-week wait in a scenario depicting hypertensive emergency, which was treated as inattentive responding. These were quality-control exclusions and not exclusions of unconventional clinical reasoning; any physician who responded differently across scenarios was kept, whatever the pattern of their decisions relative to peers. After exclusions, 555 clinical decisions from 37 physicians were available for analysis.

### Statistical analysis

We set out to quantify the influence of a small number of candidate variables on follow-up timing. The candidate variables, all varied by construction across scenarios, were the diagnostic systolic BP reading (dBP), the mean of the seven follow-up home BP measurements (fBP), the variability of the follow-up measurements (sd_fBP), an indicator for whether the patient's BP had improved relative to diagnosis (improving), and the physician's professional category (consultant). A non-linear effect of fBP was anticipated because guidelines target systolic BP below 140 mmHg but caution against values below 120 mmHg (2). We therefore included a hinge term, ΔfBP, equal to 0 when fBP is below 140 and equal to fBP minus 140 otherwise. This lets the effect of fBP change above the 140 mmHg threshold. An interaction between consultant status and ΔfBP was included to allow the slope above threshold to differ between consultants and non-consultants.

The final model was a linear mixed-effects model with a random intercept by physician:

$$Y_{ij}=\beta_{0}+\beta_{1}\cdot \mathrm{fBP}+\beta_{2}\cdot \Delta \mathrm{fBP}+\beta_{3}\cdot \mathrm{dBP}+\beta_{4}\cdot \mathrm{consultant}+\beta_{5}\cdot (\mathrm{consultant}\;\mathrm{times}\Delta \mathrm{fBP})+u_{i}+\varepsilon_{ij}$$

where *Y*_ij_ is the scheduling choice (weeks until follow-up) made by physician *i* for scenario *j*; fBP is the mean follow-up home BP; ΔfBP is the hinge term defined above; dBP is the diagnostic BP; consultant is an indicator (1 = consultant, 0 = non-consultant); *u_i_* is the physician random intercept; and *ε_ij_* is the residual. The sd_fBP and improving variables were considered as candidate covariates but did not improve model fit and are not in the reported specification.

The model was fitted in R using the nlme package (16). Model fit was assessed using the Pearson correlation between predicted and stated scheduling decisions on the same data used for fitting. This is an in-sample measure of goodness-of-fit and not out-of-sample predictive validity, which would require an independent test set. We did not perform cross-validation in this study and treat the resulting estimates accordingly.

## Results

### Model performance

Within the analysed sample, model predictions correlated with stated decisions at Pearson *r* = 0.83, with a median absolute difference of 0.1 weeks (under 1 day) between prediction and stated preference. We read this as evidence that the chosen variables capture most of the variance explainable by the scenario design, not as evidence of validity in real practice. Fit was strongest at the extremes of the fBP range (near 120 mmHg and above 180 mmHg) and weakest in the mid-range (about 150–170 mmHg), where individual scheduling decisions showed greater dispersion than the linear specification predicts. The aggregate Pearson r therefore over-represents how well the model captures the middle of the BP range, and the two-piece linear specification should be read as a parsimonious account of a relationship that has some additional curvature in that region.

### Drivers of scheduling decisions

The fitted model shows opposing trends on either side of the 140 mmHg threshold. Below the threshold, when the follow-up BP is 120 mmHg, consultants would see the patient at roughly 4 weeks (3.9 weeks; 95% CI 3.1–4.7; *P* < .001). As fBP rises toward 140, follow-up is pushed slightly later (0.05 weeks per 1 mmHg increase; 95% CI 0.01–0.09; *P* = .007), consistent with concern about overtreatment when BP is well below target. Above the threshold, higher BP shortens the interval (0.13 weeks earlier per 1 mmHg increase; 95% CI 0.09–0.17; *P* < .001).

The original diagnostic BP reading had minimal influence on scheduling (0.01 weeks sooner per 1 mmHg increase; 95% CI −0.02 to 0; *P* = .055). Clinicians appear to weight recent home BP data more heavily than the historical reading.

### Variation between clinicians

**Figure 2:**
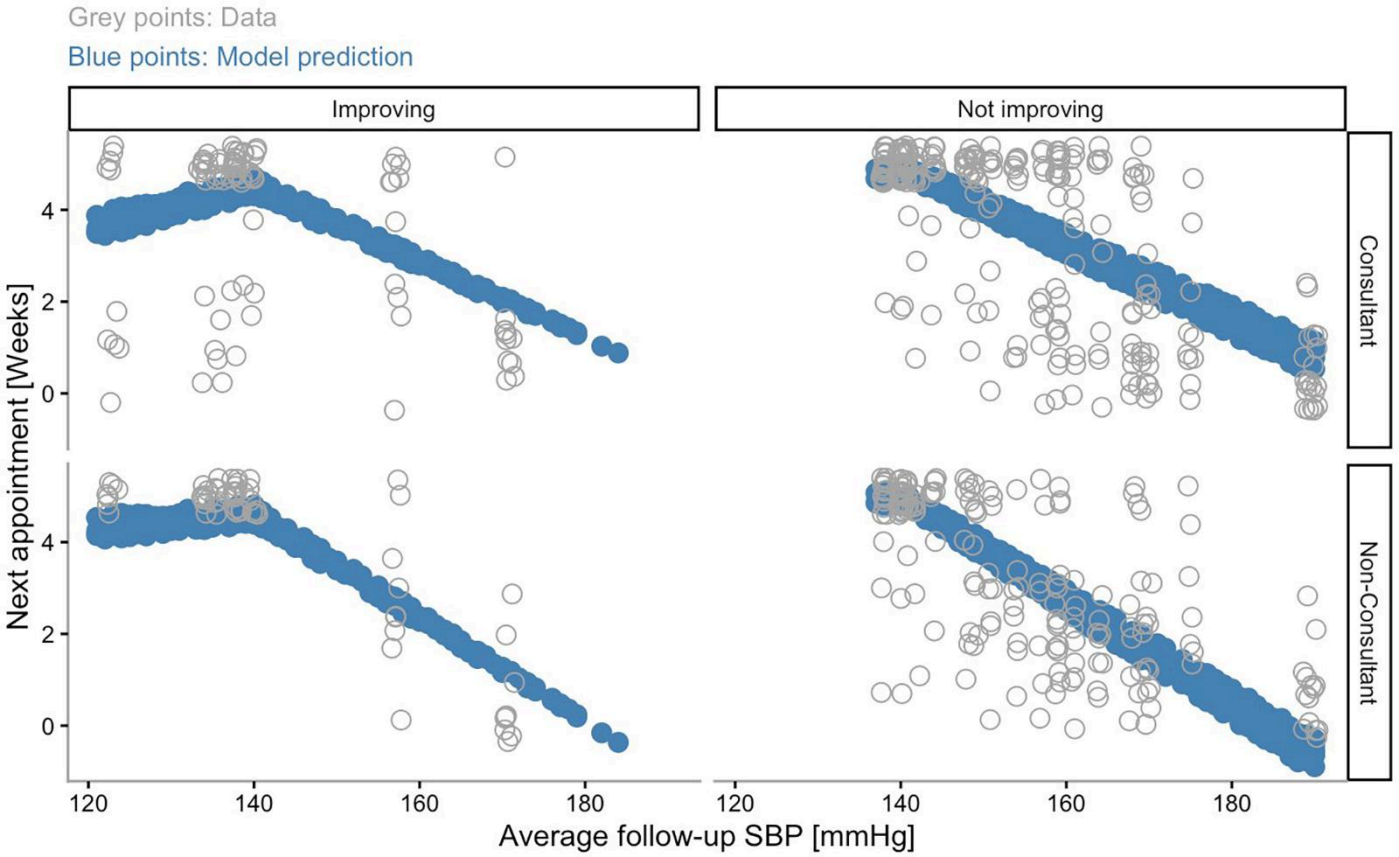
Agreement between elicited scheduling decisions (grey points, observed data) and model predictions (blue points), stratified by improvement trajectory (columns) and consultant status (rows). The *x*-axis is the average follow-up systolic BP and the *y*-axis is the chosen interval to the next appointment in weeks. Predicted values can fall slightly below zero where the model extrapolates beyond the “see immediately” option; these are read as a request for review at the earliest opportunity.

There were systematic differences between consultants and non-consultants. At a follow-up BP of 120 mmHg, non-consultants scheduled review about half a week later than consultants (0.7 weeks; 95% CI 0.1–1.3; *P* = .04). At higher BP levels, non-consultants brought review forward more steeply than consultants (0.03 weeks earlier per 1 mmHg increase; 95% CI −0.05 to −0.01; *P* = .004). The same simulated patient could therefore receive different follow-up timing depending on which clinician reviewed the data. Figure 2 shows the agreement between elicited decisions and model predictions, stratified by consultant status and improvement trajectory.

## Discussion

### Principal findings

In simulated scenarios, physicians appear to base follow-up timing on a small number of interpretable cues, chiefly the most recent home BP reading relative to the 140 mmHg threshold. A parsimonious linear mixed-effects model reproduced the elicited decisions with a high in-sample correlation. We stress that this is internal goodness-of-fit. The model has not been tested against held-out responses, against real scheduling decisions, or against patient outcomes, and generalisation to real practice remains untested. The findings are hypothesis-generating.

What the model captures is best described as clinicians' stated preferences for follow-up timing under simplified conditions. It does not describe current practice, which is shaped by clinic templates, slot availability, patient travel, and other constraints that the vignette format deliberately removed. It does not describe optimal care, which would require evidence linking scheduling intervals to outcomes, which we did not collect. The signal sits closer to what clinicians would want, all else being equal, than to either what clinicians do or what is best for patients.

### Relationship to other decision-support work

The approach has parallels in other non-communicable diseases. Risk-stratified follow-up scheduling has been described in chronic kidney disease (14), and glucose-driven pathways using continuous monitoring or HbA1c have been used to vary review frequency in diabetes (15). Telemonitoring-based decision support has been evaluated in heart failure and chronic respiratory disease. The common thread is the use of a small set of recent physiological signals to adjust the timing of clinical contact. The specific decision rules do not transfer between diseases without re-fitting, because the relevant thresholds and the clinical meaning of a given value differ by condition. Our contribution is to make the hypertension-specific rules explicit and to quantify the variation between clinicians.

### Implications for mHealth decision support

If the rules elicited here can be validated against real decisions, they could inform a scheduling aid that proposes a follow-up interval from home BP data and flags patients for earlier review. We describe this as a methodological scaffold rather than a clinical tool. Any deployable system would need to accommodate the patient’s own preferences about the timing and modality of review, whether in-person, virtual, or asynchronous, since shared decision-making increasingly defines good chronic disease care and is not represented in our data. Before any claim of clinical utility could be made, the rules would need cross-validation, external validation against real scheduling decisions, and prospective evaluation against outcomes and workload.

### Limitations

The principal limitation is ecological. The scenarios were simulated, not real consultations, and stated preferences may not match real-world behaviour. The scenarios presented a deliberately narrow view of the patient, with no comorbidity, no medication tolerability information, no prior treatment history, no patient values or preferences, and no service constraints such as clinic capacity or social context. Real scheduling decisions integrate all of these. Shared decision-making about the timing and modality of review is not represented in our data. The heuristics we describe should be read as the scheduling component that clinicians apply to BP data in the absence of other constraints, not as a complete model of how follow-up is decided.

Several further limitations apply. The online format and the absence of organisational constraints may have shaped responses in either direction; the prompt framed the task as abstract, but respondents' internalised practical considerations are likely to have influenced their answers even so. The sample was drawn from physicians in Ireland, and heuristics may differ in other health systems with different service models or guideline thresholds. The sample size, while sufficient for the chosen mixed-effects model, was modest, and we did not stratify by specialty in detail. The two-piece linear specification under-represents curvature in the mid-range of fBP, so the model summary statistics are likely to be over-confident about fit in that region. Model performance reflects in-sample goodness-of-fit only; we did not cross-validate, and the model has not been tested against real scheduling decisions or against patient outcomes. We have therefore avoided framing the model as predictive or as generalisable.

### Future directions

Several next steps follow. Replication with larger and more diverse samples, including physicians from other health systems, would test whether the same heuristics emerge. Formal cross-validation against held-out responses, and external validation against real scheduling decisions extracted from electronic health records, would be needed before any decision-support claim could be made. Prospective evaluation comparing algorithm-assisted scheduling with usual care, on clinical outcomes, workload, and patient acceptability, is the appropriate route to clinical utility. The same elicitation approach could be applied to other chronic conditions amenable to remote monitoring, such as diabetes [using continuous glucose monitoring or HbA1c (15)] and heart failure, where similar decision rules may operate.

## Conclusions

In simulated scenarios of newly diagnosed hypertension, clinicians appear to rely on a small set of interpretable cues, primarily the recent home BP reading relative to 140 mmHg, when stating their preferences for follow-up timing. The cues can be captured by a parsimonious linear mixed-effects model with good in-sample goodness-of-fit, and the model reveals systematic differences between consultants and non-consultants. Because the scenarios omitted important clinical and contextual information, and because the model has not been validated out of sample or against real outcomes, the findings are best treated as a methodological foundation for future work on mHealth-supported scheduling rather than as a clinical tool ready for deployment.

## Data Availability

The raw data supporting the conclusions of this article will be made available by the authors, without undue reservation.

## Supplementary material

The Supplementary Material for this article can be found online at: https://www.frontiersin.org/articles/10.3389/fdgth.2026.1858572/full#supplementary-material

Supplementary Figure S1 The complete set of 15 simulated home blood pressure scenarios presented to physicians, each showing the initial diagnostic reading and seven consecutive daily home BP measurements.
