# Supplementary material for: Dynamic triage in hypertension: a quantitative framework for follow-up scheduling based on clinician heuristics and home blood pressure data
Source: Front Digit Health. 2026 Jul 21;8:1858572. doi: 10.3389/fdgth.2026.1858572 (PMC13433855; doi:10.3389/fdgth.2026.1858572)
Supplement: Supplementary file 1 [file Image1.pdf]

Supplementary Figure S1

The complete set of simulated scenarios presented to physicians. In each panel the brown point is the initial diagnostic blood pressure reading and the blue points are the seven consecutive daily home blood pressure measurements recorded two weeks after diagnosis and treatment initiation. The dotted vertical line marks the day of the scheduling decision and the dashed line marks the scheduled follow-up appointment. Each caption gives the diagnostic value (dBP) and the mean of the seven follow-up measurements (mean fBP).

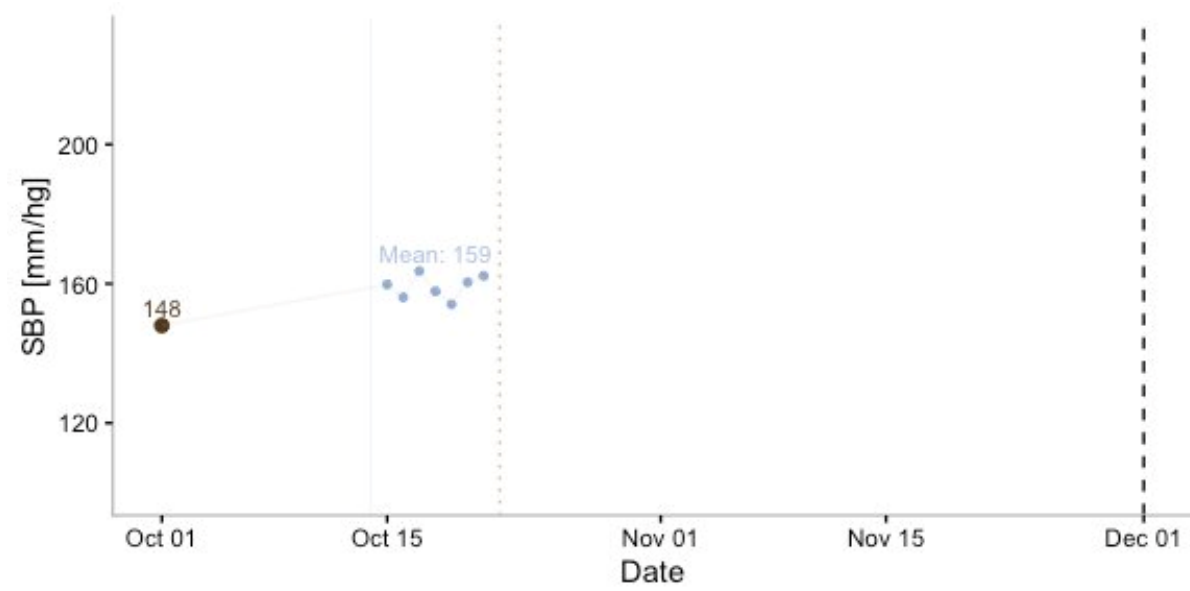

Scenario 1 (dBP 148, mean fBP 159 mmHg)

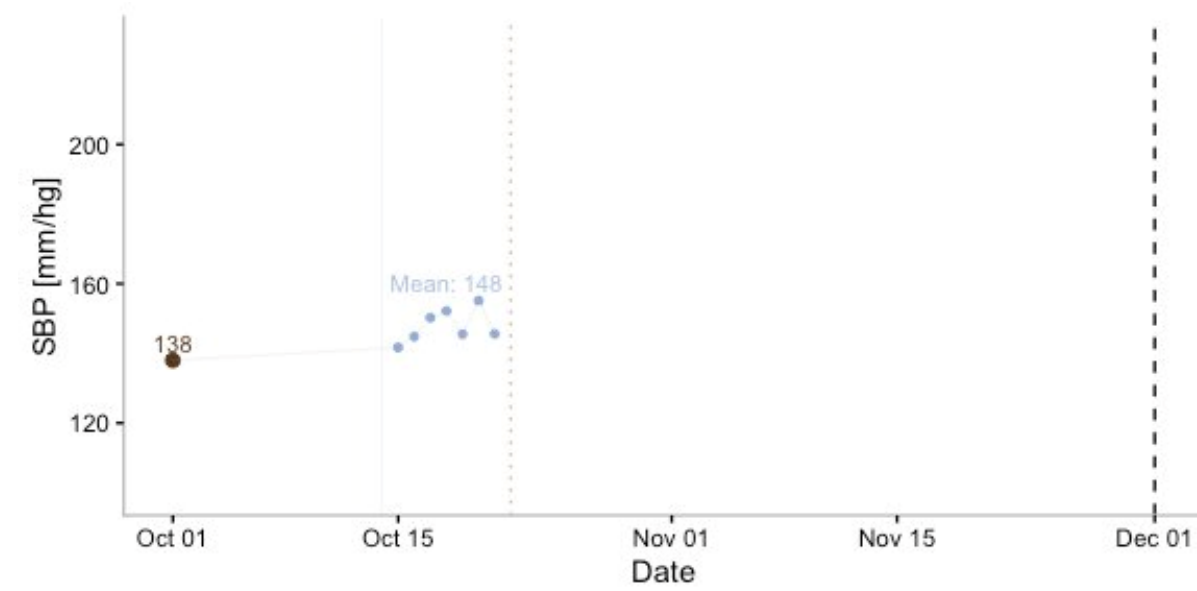

Scenario 2 (dBP 138, mean fBP 148 mmHg)

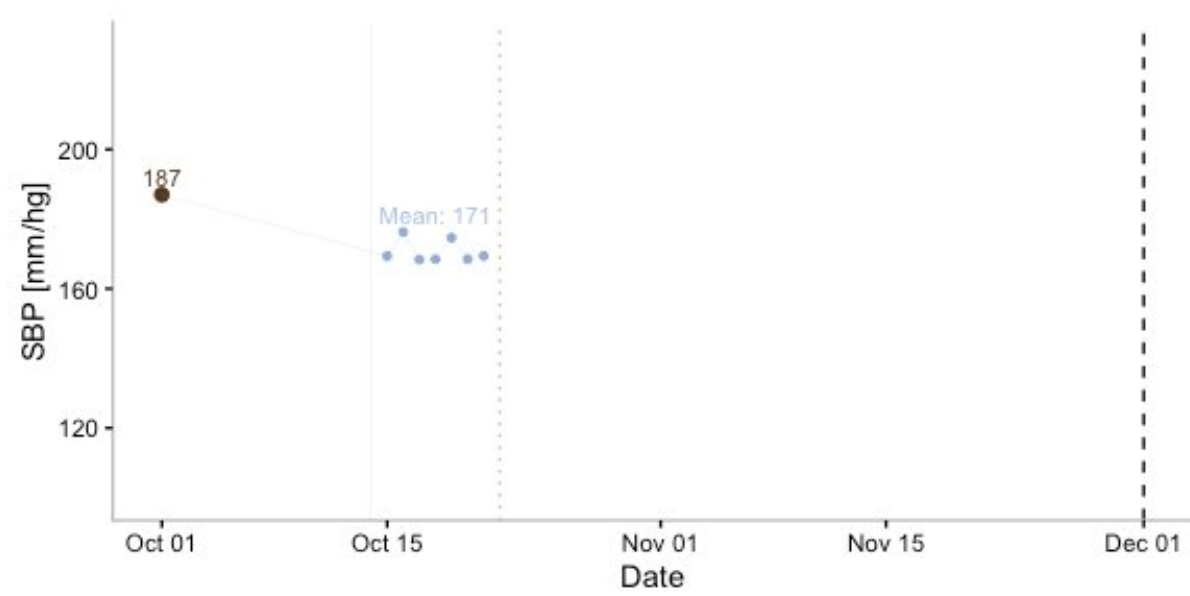

Scenario 3 (dBP 187, mean fBP 171 mmHg)

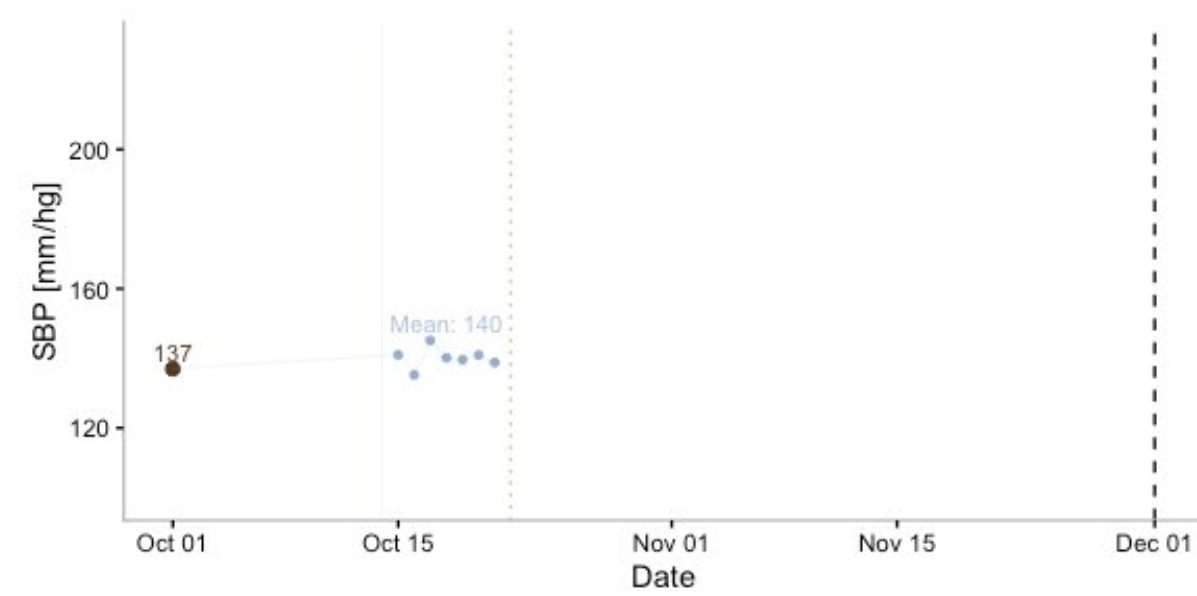

Scenario 4 (dBP 137, mean fBP 140 mmHg)

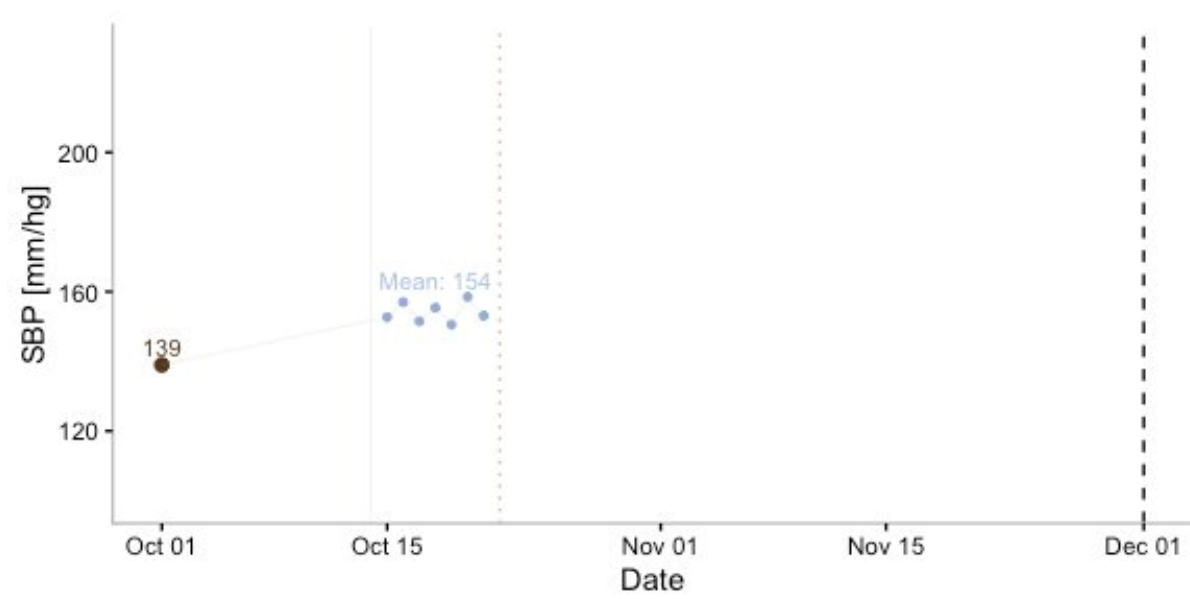

Scenario 5 (dBP 139, mean fBP 154 mmHg)

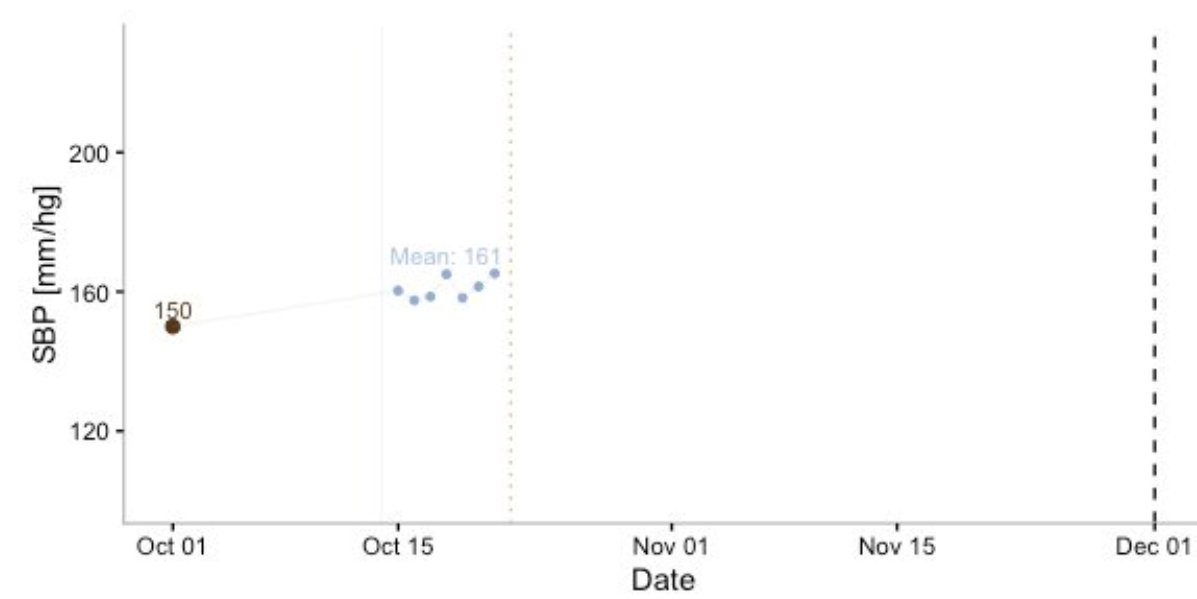

Scenario 6 (dBP 150, mean fBP 161 mmHg)

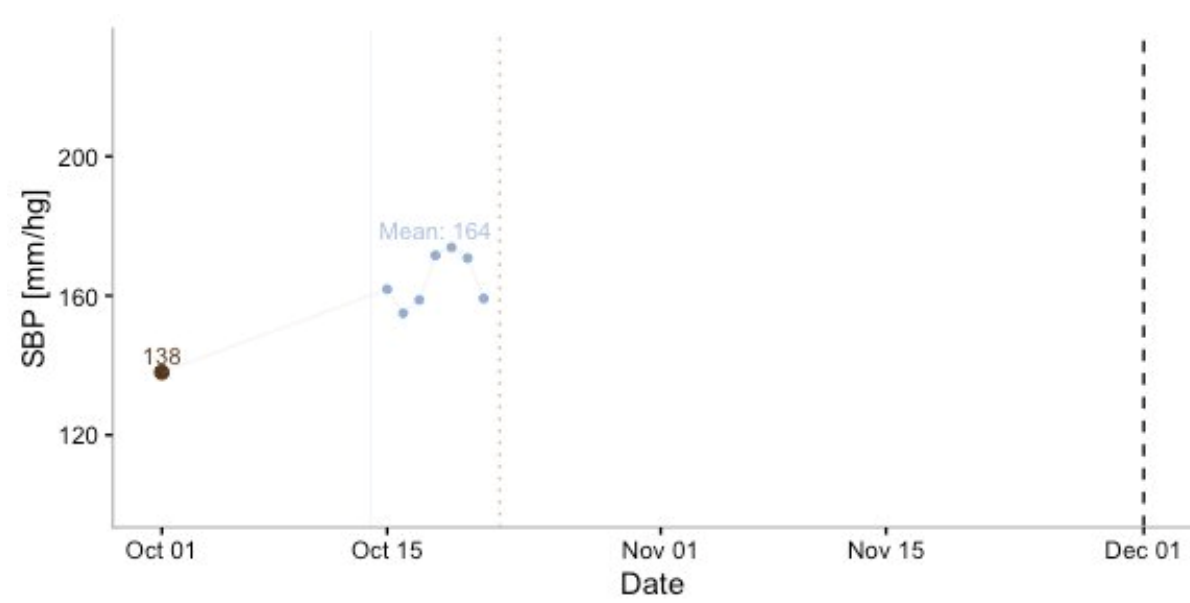

Scenario 7 (dBP 138, mean fBP 164 mmHg)

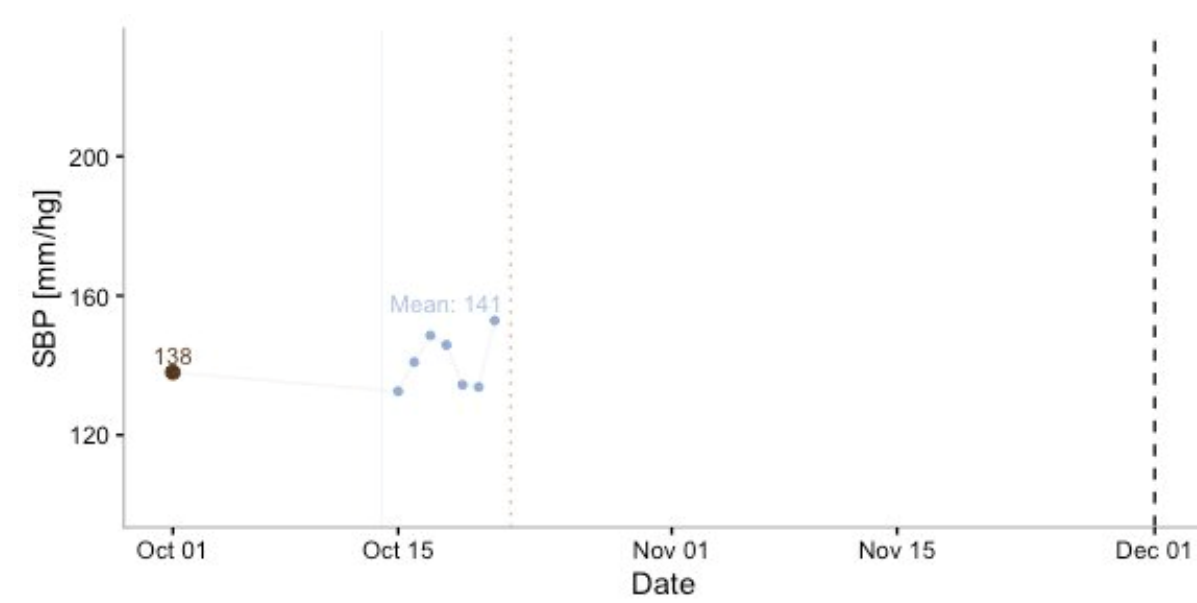

Scenario 8 (dBP 138, mean fBP 141 mmHg)

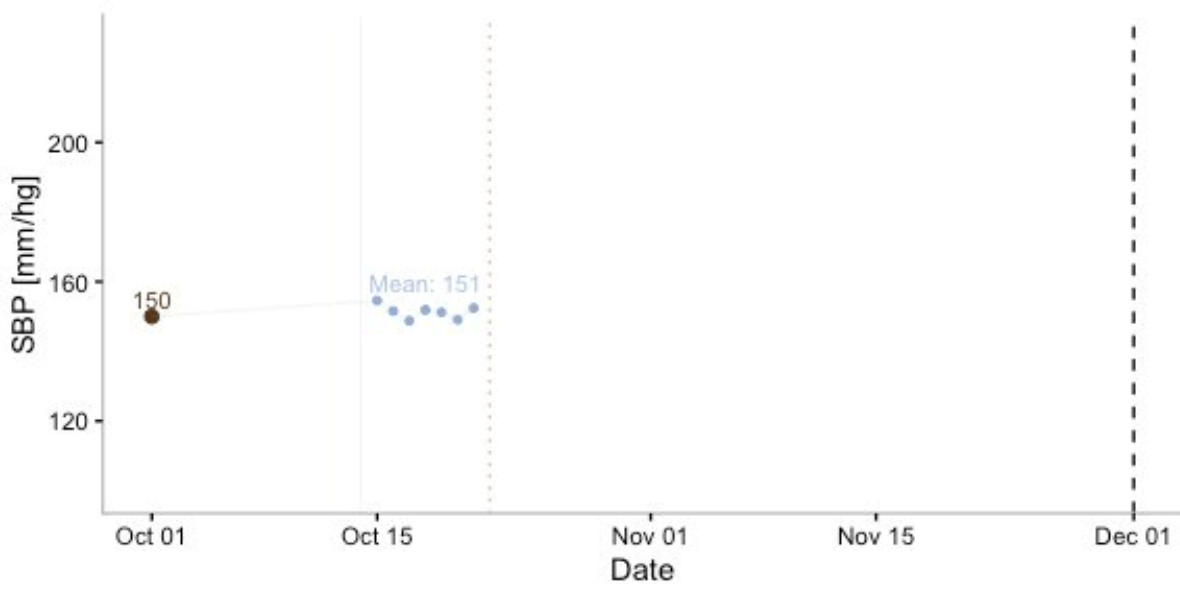

Scenario 9 (dBP 150, mean fBP 151 mmHg)

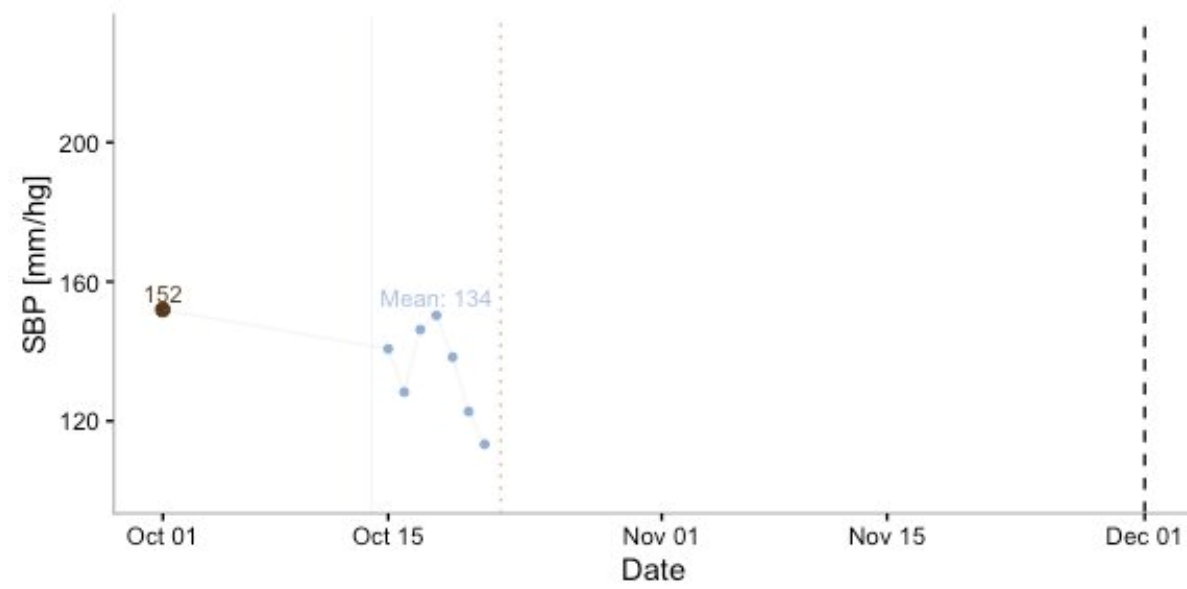

Scenario 10 (dBP 152, mean fBP 134 mmHg)

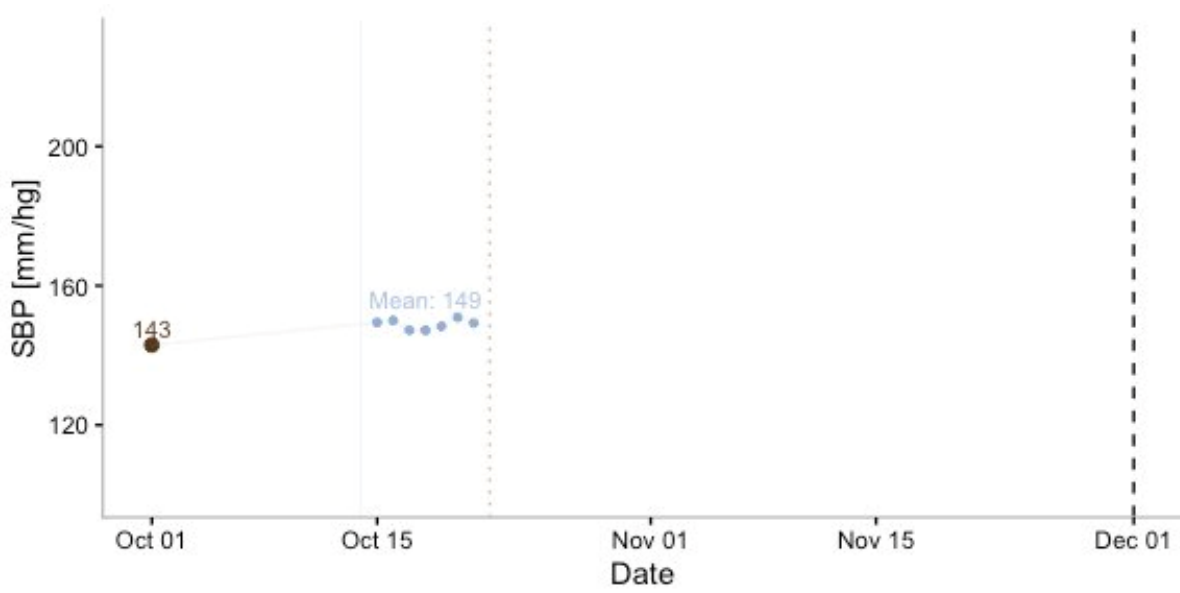

Scenario 11 (dBP 143, mean fBP 149 mmHg)

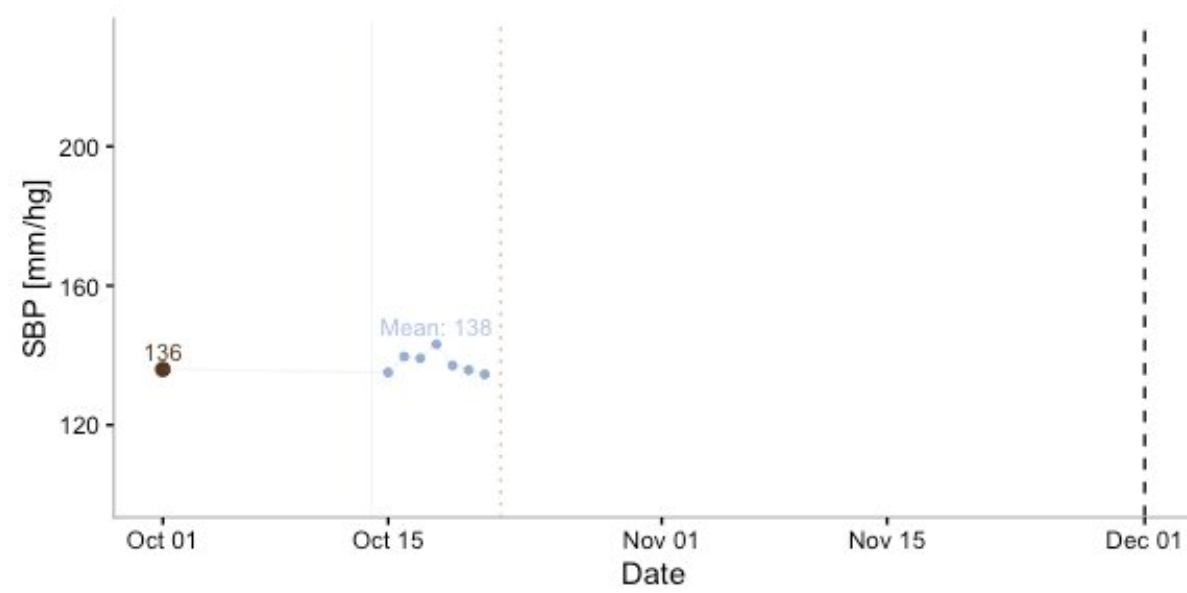

Scenario 12 (dBP 136, mean fBP 138 mmHg)

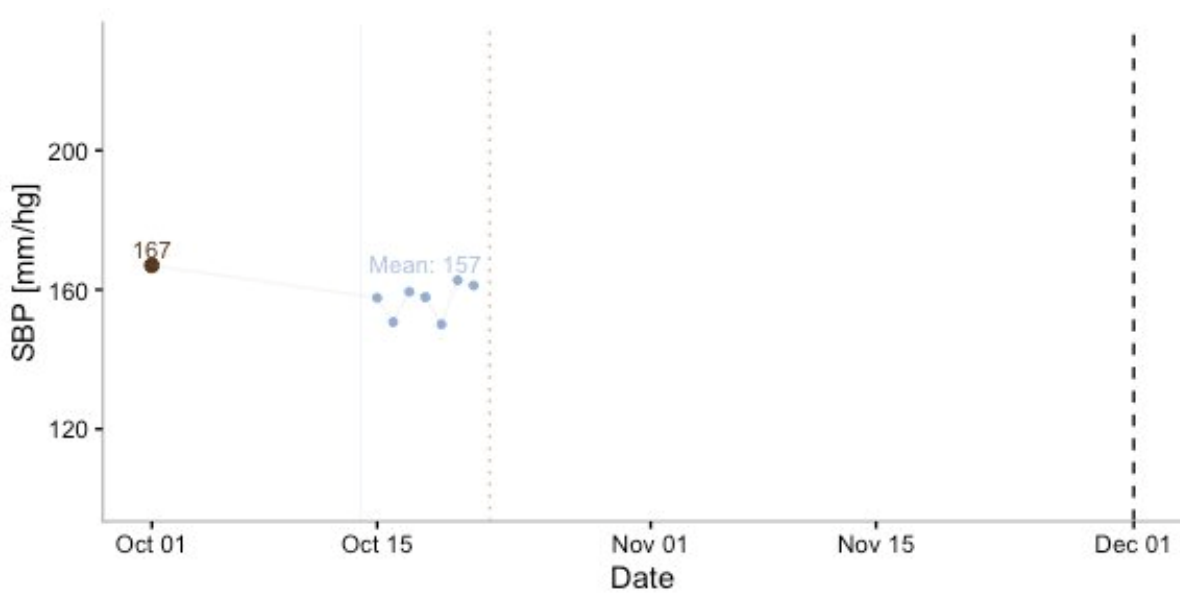

Scenario 13 (dBP 167, mean fBP 157 mmHg)

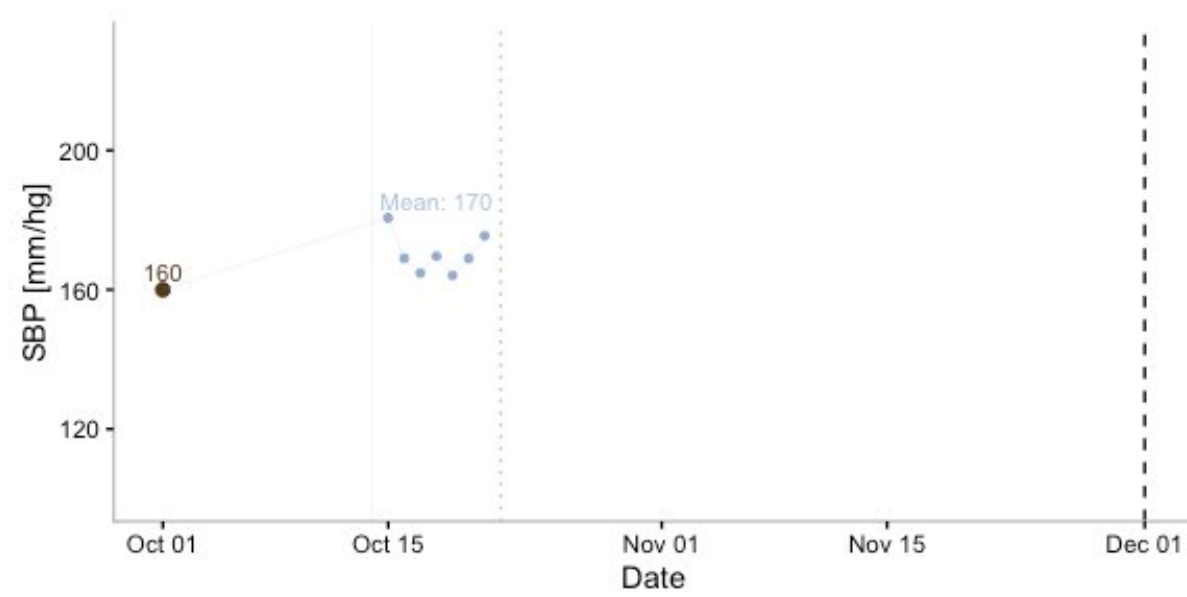

Scenario 14 (dBP 160, mean fBP 170 mmHg)

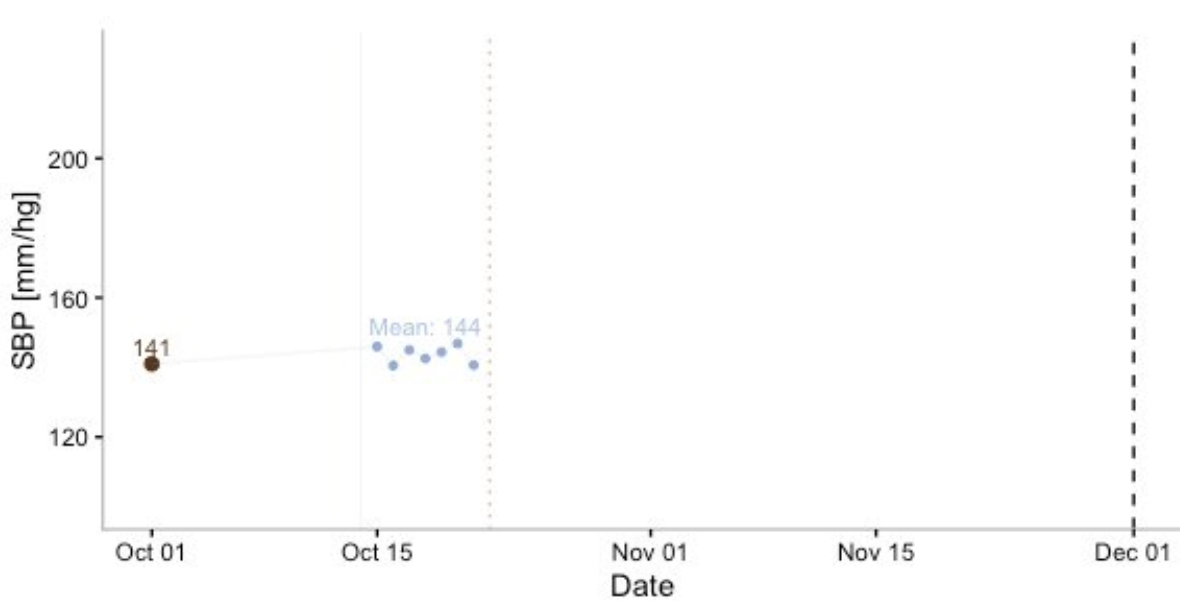

Scenario 15 (dBP 141, mean fBP 144 mmHg)
